# Returning individual research results for genome sequences of pancreatic cancer

**DOI:** 10.1186/gm558

**Published:** 2014-05-29

**Authors:** Amber L Johns, David K Miller, Skye H Simpson, Anthony J Gill, Karin S Kassahn, Jeremy L Humphris, Jaswinder S Samra, Katherine Tucker, Lesley Andrews, David K Chang, Nicola Waddell, Marina Pajic, John V Pearson, Sean M Grimmond, Andrew V Biankin, Nikolajs Zeps

**Affiliations:** 1 Cancer Research Program, Garvan Institute of Medical Research, the Kinghorn Cancer Centre, Sydney, NSW, 2010, Australia; 2 St John of God Subiaco, Perth, WA, 6008, Australia; 3 School of Surgery, The University of Western Australia, Perth, WA, 6009, Australia; 4 Department of Anatomical Pathology, Royal North Shore Hospital, Sydney Australia and University of Sydney, Sydney, NSW, 2065, Australia; 5 Department of Surgery, Royal North Shore Hospital, Sydney, NSW, 2065, Australia; 6 Queensland Centre for Medical Genomics, Institute for Molecular Biosciences, University of Queensland, St Lucia, QLD, 4072, Australia; 7 Hereditary Cancer Clinic, Prince of Wales Hospital, Randwick, Sydney, NSW, 2031, Australia; 8 Genetic and Molecular Pathology, SA Pathology, Women's and Children's Hospital, North Adelaide, SA, 5006, Australia; 9 Wolfson Wohl Cancer Research Centre, Institute of Cancer Sciences, University of Glasgow, Glasgow, Scotland, UK; 10 West of Scotland Pancreatic Unit, Glasgow Royal Infirmary, Glasgow, Scotland, UK; 11 Department of Surgery, Bankstown Hospital, Eldridge Road, Bankstown, Sydney, NSW 2200, Australia; 12 South Western Sydney Clinical School, Faculty of Medicine, University of New South Wales, Sydney, NSW, 2170, Australia; 13 Wolfson Wohl Cancer Research Centre, Institute of Cancer Sciences, University of Glasgow, Garscube Estate, Switchback Road, Bearsden, Glasgow, Scotland G61 1BD, UK; 14 Garvan Institute of Medical Research, 370 Victoria St, Darlinghurst, NSW, 2010, Australia

## Abstract

**Background:**

Disclosure of individual results to participants in genomic research is a complex and contentious issue. There are many existing commentaries and opinion pieces on the topic, but little empirical data concerning actual cases describing how individual results have been returned. Thus, the real life risks and benefits of disclosing individual research results to participants are rarely if ever presented as part of this debate.

**Methods:**

The Australian Pancreatic Cancer Genome Initiative (APGI) is an Australian contribution to the International Cancer Genome Consortium (ICGC), that involves prospective sequencing of tumor and normal genomes of study participants with pancreatic cancer in Australia. We present three examples that illustrate different facets of how research results may arise, and how they may be returned to individuals within an ethically defensible and clinically practical framework. This framework includes the necessary elements identified by others including consent, determination of the significance of results and which to return, delineation of the responsibility for communication and the clinical pathway for managing the consequences of returning results.

**Results:**

Of 285 recruited patients, we returned results to a total of 25 with no adverse events to date. These included four that were classified as medically actionable, nine as clinically significant and eight that were returned at the request of the treating clinician. Case studies presented depict instances where research results impacted on cancer susceptibility, current treatment and diagnosis, and illustrate key practical challenges of developing an effective framework.

**Conclusions:**

We suggest that return of individual results is both feasible and ethically defensible but only within the context of a robust framework that involves a close relationship between researchers and clinicians.

## Background

It is generally agreed in national and international ethical guidelines that, broadly, the results of research should be made available to participants [1,2] and this is echoed in the literature on this topic [3,4]. This has generally been restricted to aggregate data that are made available through written publications such as journal articles or through research websites. Individuals are not identifiable in such aggregate reports, nor is information relevant to an individual usually reported back to participants, although arguments have been made that individual results should be made available in some circumstances [5-7].

Since the first draft of the sequence of the human genome [8,9] there has been a literal revolution in the way in which genomic sequencing is performed. Over the past decade, next-generation sequencing (NGS) has developed to the point that rapid and relatively affordable sequencing of individual genomes is a reality [10]. NGS offers the promise of tremendous public benefit as it underpins both improvements in our understanding of disease as well as substantially changing our practical ability to translate this knowledge through improved diagnostics and therapeutics. The advent of NGS has also seen an increase in the debate concerning the disclosure of individual results of genomic data to research participants.

Knoppers *et al*. [11] previously commented that returning individual results appears to be contrary to the fundamental purpose of research, but noted that if this was to occur, it should be addressed in consent documentation prior to participation. Similarly, Forsberg *et al*. [12] argued against the return of results from research on the basis that it is a collective effort to improve public health and that this could be seen as a shift from autonomy and individual rights to collective responsibility and solidarity. Nevertheless some have argued that there may be an obligation to return individual results, and Beskow and Burke [13] proposed a model based on an ancillary care framework as the basis for evaluating when such obligations may arise. Similarly, Ravitsky and Wilfond [14] suggest that different results may require different decisions even within the same study, and Renegar *et al.*[15] describe a benefit-to-risk assessment to balance the potential positive versus negative consequences to participants. Fabsitz *et al.*[16] outlined five principles for determining if research results should be returned to individuals and these are echoed by Amy McGuire and colleagues’ [17] recommendations. In brief, these identify a need to obtain appropriately informed consent, to ensure that any results are analytically valid, that there is potential for intervention based on the results, that there are mechanisms in place to return results, and that adequate resources exist to do so.

These commentaries have permitted researchers to decide primarily not to return results for practical reasons; however, Wolf *et al.*[6] recently argued that there is a responsibility to establish frameworks for return of individual results as a core component of any research. In contrast, Bledsoe *et al.*[18] have proposed that context is important, and rather than a generalized obligation, careful evaluation based upon the type of research and likely clinical utility be considered. The American College of Medical Genetics has recently suggested that the use of NGS in clinical practice may give rise to an obligation to actively search for 57 (revised to 56) genes linked to 24 genetic conditions [19]. Whilst this is only a recommendation for clinical practice (with a disclaimer), policy makers are noting these documents and there is the potential for this to become a more general obligation for both clinical practice and research. The Presidential Commission for the Study of Bioethical Issues, with the Office of the Secretary, Department of Health and Human Services in the United States of America are currently reviewing this approach.

This has become an issue of global importance because projects like the International Cancer Genome Consortium (ICGC) and the international Rare Diseases Consortium (IRDiRC) involve the exchange of genetic data across many borders [20]. Indeed, the creation of large-scale genomic data repositories available to external researchers implies that there are no borders when it comes to these data, and therefore international harmonization of guidelines will prove to be vital. Statements by the 1991 Council for International Organizations of Medical Sciences (CIOMS) [21] and the Council of Europe [22] already suggest that it may be reasonable to return individual information arising from genomic studies in specific contexts.

An ethical obligation to establish a plan for returning potentially relevant findings exists within the Australian National Statement on Ethical Conduct in Human Research [2]. Researchers are required to consider the likelihood of making any such findings and to have an ‘ethically defensible plan’ to withhold or disclose these. Whatever the plan, there is an obligation to notify potential research participants about the risks involved during the consent process and to have appropriate mechanisms that will handle return of any individual results as they arise.

Whilst the published reports and opinions are helpful, to the best of our knowledge there appears to be few empirical data on or evaluations of actual situations involving return of research results. In this article we present data from The Australian Pancreatic Cancer Genome Initiative (APGI), an Australian contribution to the ICGC, where individual research results were returned to participants and the impact and challenges of this process explored. We present the framework for which return of results has been constituted and provide an overview of case studies where research data were returned. We use these examples to illustrate how our framework worked in practice, discuss the ethical and practical challenges encountered, and make recommendations based on our experiences.

## Methods

### Study population

The APGI [23] is a multi-disciplinary research network in Australia, with over 100 active contributors. The goal of the APGI is to comprehensively catalogue the genomic abnormalities in 375 prospectively recruited pancreatic cancer patients by high-throughput NGS [24]. The study includes recruitment of participants undergoing surgical treatment for pancreatic cancer, collection of tissue and blood specimens and subsequent sequencing of the entire genomes of tumor and matched normal DNA, and profiling of gene expression and methylation states [25,26]. Ethical approval was obtained from the human research ethics committee at each participating institution, conducted in accordance with the National Statement on Ethical Conduct in Human Research (2007) and the Declaration of Helsinki (Additional file 1). All participants provided written informed consent upon entry to the study, which included their preference with respect to return of results. Additionally, written informed consent was obtained from the next of kin for publication of the three cases and a copy of the consent form is available to the Editor on request.

### Establishing an ethically defensible and clinically practical framework - the ethically defensible plan

The Australian National Statement on ethical conduct in Human Research (2007) section 3.5.1 states 'Where research may discover or generate information of potential importance to the future health of participants, or their blood relatives, researchers must prepare and follow an ethically defensible plan to disclose or withhold that information'. What is key about this guideline is that it does not assume an obligation to return results but in essence enacts Beskow and Burke’s [13] proposal to establish context-dependent mechanisms for when and how research results should be returned.

Given the obligation in Australia to consider this guideline, the framework set out by the APGI for returning results employs a context-dependent approach, and enacts a category-based system for the characterization of research findings as previously proposed [27]. The framework was developed as an iterative, evidence-based and consensus driven process, with engagement of key stakeholders within the APGI, including surgeons, oncologists, scientists, ethicists and clinical geneticists. Importantly, the plan is reviewed annually, to keep pace with advances in technology and emerging reference data. The central components of the framework are informed consent, analytical validity, clinical relevance or significance of the finding, communicability and delivery of results (Additional file 2).

### Consent

Through the consent process, participants are provided with information in relation to the fact that relevant findings may be discovered in the course of the study, and that these may not be limited to pancreatic cancer. The consent process allows participants the choice to 'opt in' to have individual results communicated, and given the short survival of many individuals with pancreatic cancer, the choice of to whom else they may be communicated. It is usual practice during the consent process to obtain contact details of a family member or significant other who is identified to receive information (Additional file 3). Whilst participation of family members in the consent process is typical and was encouraged for the above reasons, relatives are not considered formal participants of the study and explicit consent was not required under this protocol. Participants are informed that any information would initially be discussed with their treating doctor, and their preference for returning results is logged and tracked through a research database.

### Significance of findings

We focused on cancer-related incidental findings and segregated our data into three categories of significance based on available evidence [27,28] and clinical consensus amongst APGI clinical specialists (Table 1). Whilst there is a great deal of debate about where one may draw the line between categories and which genes may fall into each [19], we made a practical, project-directed decision, where findings were related to the research indication.

**Table 1:** Categories of significance and evidence and examples of genes and pathways included in the various categories felt to be specific to this protocol

**Category**	**Key criteria**	**Examples of genes or pathways**	**Justification and evidence**
Medically actionable	Analytically validated assay	Highly penetrant variants in cancer-related genes associated with disorders	Certified and established clinical or practice guidelines
	Clinical validity	*ATM*	Direct clinical utility of a diagnostic or therapeutic nature that is part of accepted clinical practice
	Direct clinical utility (established guidelines with regard to prevention, diagnosis, prognostication and/or therapy)	*MLH1, MSH6, MSH2 PMS2*	Preventable disease due to established treatment approaches
		*APC*	Diagnostic grade assay available in standard clinical practice
		*BRCA1 or BRCA2*	
		*PALB2*	
		*MUTYH*	
		*VHL*	
		*MEN1, RET*	
		*NF2*	
		*STK11*	
		*TP53*	
Potential clinical utility	Clinical validity	*ERBB2* (*HER 2/neu*) amplification [29]	Scientific literature (high level of evidence)
	Clinical utility not proven in current treatment setting	Defects in genes involved in homologous recombination [30]	Pre-clinical evidence
	Availability of clinical trials specific to the finding	Mutations of *EGFR, KIT, BRAF, BRCA1/2, PALB2*, where therapeutics are potentially accessible and clinically appropriate	Clinical trial signals
		Wild-type *KRAS*[31]	Therapeutic opportunity:
			Drug repurposing
			Rescuing therapeutics
			Clinical trial availability
			Diagnostic grade assay available
			Potentially highly replicable robust single laboratory research assay
Undetermined significance	Variants that have not as yet been definitively linked to a phenotype, clinical outcome or intervention	Not applicable	These variants form the basis of future research

### Communication strategy

Communicability considers the practicality of communicating results, the circumstances of the participant and treating clinician, while delivery of results considers how best the results could be communicated. In the APGI study, participants are initially contacted in writing, and invited to contact the research team via telephone. They are given the option of receiving the information face-to-face, which, in our opinion, is the best method of delivery in sensitive situations. Wherever allowable, results are to be communicated to the clinical care provider or treating medical team for their consideration. At times the delivery will take place face-to-face in a multidisciplinary setting, with the participant, family, clinicians and researchers present. Contact information of participants is collected via an interview performed at the time informed consent is obtained.

## Results

Since June 2009, 556 participants have been enrolled as part of the APGI. As with previous studies [32,33], our population was very willing to be contacted, with 95% (n = 530) indicating through the consent process that they would like to be contacted and notified about important research information. Furthermore, 100% of those that agreed to be contacted (n = 530) also agreed to provide contacts for a family member or significant other should they not be available to receive the information.

### Variant calling

SNPs were identified with a dual-calling strategy using qSNP [34] and GATK [35], while small insertions and deletions (indels) were called with Pindel [36]. Variants that were specific to the tumor sample with no evidence in the matched germline sample were considered somatic, while those present in the germline sample and the tumor sample or specific to the germline sample were classified as germline. Germline SNPs identified by GATK and/or qSNP were marked as 'PASS' if they contained at least five sequence reads containing the variant with a minimum of four novel starts. If a germline SNP was detected by both GATK and qSNP, it was considered high-confidence. Germline indels were filtered to include those with a minimum of three novel starts. All germline variants were annotated using ENSEMBL v70 and dbSNP130 and those in genes of interest underwent manual inspection using IGV [37]. Genes that were specifically focused on due to practical actionability are outlined in Table 1. In addition, unusual or outlier mutational signatures were also examined.

High-confidence germline calls in candidate genes were further annotated with standard tools [38] assessing the amino acid sequence change, minor allele frequency and predicted impact on protein function. All frameshift indels, truncating single-nucleotide variants and missense single-nucleotide variants that were deemed possibly damaging, or with qualitative scores indicating the finding was deleterious, were further investigated using inherited disease mutation databases [39] and literature review. Those previously reported as pathogenic with functional evidence were then sent for confirmatory testing using a diagnostic grade assay. Other variants were not considered sufficiently validated or lacked supporting evidence to return as individual results.

Of the 556 participants, 285 underwent genomic sequencing and 17 medically actionable or clinically useful findings were validated and returned (Additional file 4). In addition, clinicians participating in the study directly requested results on an additional eight participants that did not have results that were medically actionable or of potential clinical utility. Out of the 17 results that were returned, 4 represented germline susceptibility mutations, 3 had genomic changes that altered diagnosis, and 10 had potential therapeutic relevance. With the exception of one case where the diagnosis was in question, all results were confirmed in independent, diagnostic grade assays. Most results (15; 88%) were communicated to treating clinical teams or primary care providers, and 2 were directly communicated to participants or their next of kin. Twelve (70%) were actioned and resulted in an intervention, either influencing a clinical decision or prompting involvement in a screening program. No adverse events were reported with the return of results process. Our three cases were selected as representative of the types of cases we encountered, and are illustrative of the specific categories we define in this framework.

#### Case studies

Case study 1: category 1 (medically actionable finding) - inherited predisposition to cancer with relevance to other family members

##### Scenario

A 78 year old Female underwent surgery for pancreatic cancer and provided standard APGI consent.

##### Significance of finding

Germline analysis revealed the presence of a deleterious mutation in exon 11 of the *BRCA2* gene (c.5239insT). This mutation is expected to produce the *BRCA2* inherited breast cancer phenotype. This mutation confers a 49% lifetime risk of breast cancer and 18% lifetime risk for ovarian cancer [40]. To obtain independent confirmation, this result was verified in a diagnostic laboratory with an accredited assay [41].

##### Description of ethical and practical issues

The participant was deceased at the time the results became available. Following the ethically defensible plan, after discussion with a cancer geneticist, the listed next of kin, the participant’s son, was sent a letter with an invitation to contact the research team to discuss results. After receiving the letter, contact was made within one week where findings were discussed in general terms, and a referral was given to a familial cancer clinic for counseling and further testing. In the meantime, the research team communicated the specific findings to the local familial cancer clinic. This was an important part of the process and it enabled the genetic counselors to consider the specific sensitivities related to this case, and plan appropriate intake procedures. At subsequent appointments with the familial cancer clinic, the son and two daughters were identified to have inherited the *BRCA2* mutation. The participant’s son has joined a pancreatic and prostate cancer screening program at his local hospital, and his sisters have undergone risk reduction surgery. Several months later, the research team followed up with the participant directly and he expressed sincere gratitude for receiving the information. He said 'For me, knowledge is power. I am now more informed about my future health'.

##### Conclusions and recommendations

The major consideration here was the communication strategy with the next of kin given the fact that the participant was deceased and the next of kin had no overlapping or ongoing relationship with any clinical care provider. Australian privacy law [42] does not address the use of information from deceased persons. However, an important amendment to the privacy act [43] allows for communication with family members without consent of a person under their care if a medical practitioner feels there is a risk of harm to them arising from knowledge about that person. In our case, participants were given the option of identifying a contact person in the event of their death, but not all family members may have been aware of the possibility that research was being done that could generate information that could have an impact on their own health. This does highlight the importance of anticipating the possibility of important results being generated and to establish protocols to include family members in the consent process and to ensure robust pathways are in place for the delivery of results in situations such as this.

#### Case study 2: categories 1 and 2 (medically actionable and potential clinically utility) - inherited predisposition to cancer and potential implications to current therapy

##### Scenario

A 56 year old female underwent surgery for pancreatic cancer and provided standard APGI consent.

##### Significance of finding

Germline analysis revealed the presence of a deleterious mutation in exon 11 of the *BRCA2* gene (c.5410_5411delGT). This mutation would be expected to produce the *BRCA2* inherited breast cancer phenotype. Results were verified using a diagnostic grade assay in a certified laboratory, and a formal report was issued.

##### Description of specific ethical and practical issues

The participant was alive and undergoing second line treatment for metastatic disease. Results were communicated directly to the treating clinician due to immediate potential clinical utility. At the time the participant was alive and progressing on the current chemotherapy regimen. The treating medical oncologist discussed the findings with the participant, and offered the opportunity to switch to a therapy thought to be active in other cancers with this mutation [30,44].

The participant opted for the suggested change in treatment. Meanwhile, the participant was also referred to a familial cancer clinic for counseling and further testing. The participant had no siblings or children to be included in this process. The participant stated that she 'was happy hearing of this finding… and looking forward to a shot at a different treatment that might suit me better'. At last follow up, the participant was alive with stable disease on therapy.

##### Conclusions and recommendations

Although this case represents an equivalent result and expected phenotype as the previous case, it has unique and specific ethical considerations. In contrast to the previous case, the treating medical oncologist was the initial gatekeeper of the information as the participant was still alive and there were potential therapeutic implications. Due to the progressive nature of the participant’s disease, the clinical utility was deemed by the clinical teams to be the greater priority in this instance. This demonstrates that whilst two results can be categorized similarly, the circumstances and outcomes can be very different, highlighting the importance of context.

#### Case study 3: category 2 (potential clinical utility through change of diagnosis)

##### Scenario

A 32 year old female underwent biopsy for pancreatic cancer and provided standard APGI consent.

##### Significance of finding

Somatic analysis detected mutations in *BRAF* (*V600E*) with wild-type *KRAS* and loss of heterozygosity (LOH) of the *APC* locus. Polymerase chain reaction and pyrosequencing in a NATA accredited (CLIA equivalent) diagnostic laboratory confirmed wild-type *KRAS* and *BRAF* (*V600E*) mutations. This mutation pattern was consistent with a colonic rather than a pancreatic carcinoma [45]. Results were reported to the treating medical oncologist and surgeon. On receipt of new information, further investigations were undertaken, and a primary colonic adenocarcinoma was identified.

##### Description of specific ethical and practical issues

Results were communicated directly to the treating clinical team (surgeon and medical oncologist) due to potential diagnostic significance, and subsequently discussed with the participant and family. Due to the clinical implications, an early decision to expedite confirmatory testing and reporting of results was made. The prognosis for colonic carcinoma is greatly different to that of pancreatic adenocarcinoma, and influences decisions with regard to clinical management.

##### Conclusions and recommendations

Return of research results has implications beyond incidental or secondary findings related to adult onset diseases and can have immediate value. For those undertaking prospective observational studies, we would suggest that strategies be developed within a framework for returning results, specific to such situations where the stakes are high and the timeline is important.

## Discussion

This exploration of the foundations, processes and practicalities of returning research results to participants demonstrates that research results can be returned to treating clinicians and patients in a meaningful way, with positive outcomes. We demonstrate that opportunities for alternative treatment options can be introduced through this mechanism, which can ultimately benefit the individual. This is especially pertinent to rapidly fatal diseases such as pancreatic cancer where prognosis remains poor using standard approaches. This report was not intended to further stimulate the debate about whether to return research results or not, but rather to provide empirical evidence illustrating the realistic challenges and opportunities that exist.

It is relevant that mechanisms to manage these processes, such as an ethically defensible plan, are considered from the outset, formally documented and implemented in a multi-disciplinary environment. It is important that such frameworks are not devised as blanket rules, but are dynamic so that they can be adapted in response to the context of the result, the participant, the growth of reference data and changes in guidelines and policy.

These data also provide insights into participant preferences, and how these views can differ from that of researchers and ethical review boards as 95% of participants opted-in for the return of meaningful research results. Human research ethics committees are obliged under Australian ethical guidelines to review the appropriateness of plans to communicate results or not to do so, but interestingly only 3 of 14 committees acknowledged the ethically defensible plan, or made remarks on the return of results issue in any way. Further to this, we initially sought ethics committee guidance on this issue, and were met with limited direction, recommendation, references or advice. This suggests that perhaps ethical review boards lack the knowledge or expertise to guide researchers adequately on this topic. It will be important that policies and guidelines are established to guide ethics boards when advising on return of research results.

More data are required concerning operability, cost and infrastructure requirements for returning research results, but these should assess not only the cost but also the potential savings and benefits of using genomic information to guide clinical decision-making. There would be little point in developing policies to return research results but not provide the capacity or resources to do so. We highlight the importance of relationships between researchers and clinicians and participants, making communicability less troublesome and ultimately less costly. Furthermore, the preparation and coordination of disclosure was performed by a member of the research team who carries out a liaison role, and is intimately involved in communicating between the researchers, clinical teams and participants. Researchers who consider returning results should identify a member of the research team who is an effective communicator and has a clinical sciences background.

The case studies also highlight the issue of where in the care continuum obligations exist for genomic information. One can predict that results will continue to emerge throughout the life of a patient post-diagnosis, as those findings that are of unknown significance at present will presumably not always be so. Limiting the duration of obligation of researchers to return results is likely a practical necessity and should be made clear to potential participants during the informed consent process.

We acknowledge these data have limitations. The patient group is clinically homogenous and mostly under the management of specialist clinical teams. These findings may not generalize to all individuals who are asymptomatic and have no personal or family history of cancer. Nevertheless, we argue that this is perhaps the ideal setting for returning results, as it ensures that the persons responsible have a well-established relationship with the patient and also have the necessary opportunity to communicate with them.

## Conclusions

The literature is replete with commentaries regarding the risk of disclosing research findings. With the lack of real life case studies, the benefits are rarely if ever discussed. The benefits, as shown by this report, are tangible and enduring. It is prudent for funding bodies, policy makers and governments to work with research and patient communities to build an evidence base and conduct appropriate research and analysis whilst articulating appropriate guidelines and policies. Investment in the ability to return research results is not only an ethical imperative but is of fundamental importance to the translational research process, as it affirms participants as true partners in the adoption of genomic medicine.

## Abbreviations

APGI: Australian Pancreatic Cancer Genome Initiative; ICGC: International Cancer Genome Consortium; NGS: next-generation sequencing; SNP: single-nucleotide polymorphism.

## Competing interests

The authors declare that they have no competing interests.

## Authors’ contributions

ALJ, NZ, DKM, SLS, AJG, KSS, DKC, NW, MP, SMG and AVB were responsible for the concept and design of the study. All authors participated in collection and assembly of data, and ALJ, NZ, JVP, JH, and AVB were involved in data analysis and interpretation. AJG, JSS, KT, LA, AVB, DKC and APGI were involved in provision and management of patients. All authors were involved in manuscript writing and final approval of the manuscript.

## Supplementary Material

Additional file 1: Table S1 Outline of each episode of results returned. Click here for file

Additional file 2 All associated approving human research ethics committees. Click here for file

Additional file 3 APGI Return of Results Guidelines, which outline standard procedures instituted for the return of results process. Click here for file

Additional file 4 Standard language used in the Participant Information Statement. Click here for file
