# Supplementary material for: Returning individual research results for genome sequences of pancreatic cancer
Source: Genome Med. 2014 May 29;6(5):42. doi: 10.1186/gm558 (PMC4067993; doi:10.1186/gm558)
Supplement: Additional file 1: Table S1 — Outline of each episode of results returned. [file gm558-S1.docx]

**Supplementary Table 1: Detail of results returned to participants**

| Finding | Variant | | Key Criteria | Rationale | Communication | Outcome |
| --- | --- | --- | --- | --- | --- | --- |
| Category 1: Medically Actionable | | | | | | |
| Deleterious *BRCA2* germline mutation | | c.5239insT | Reported in the Breast cancer Information Core [BIC] database where it is listed as being clinically important [^1^](#_ENREF_1) Analytically validated assay Established management guidelines for prevention, diagnosis [^2^](#_ENREF_2) | confers cancer susceptibility [^3^](#_ENREF_3) | Participant deceased; member of research team (treating clinician) to next of kin | Referred to Familial Cancer Clinic, screening and risk reducing surgery undertaken by offspring confirmed positive |
| Deleterious *BRCA2* germline mutation | | c.5722_5723delCT | Reported in the Breast cancer Information Core [BIC] database where it is listed as being clinically important [^1^](#_ENREF_1) Analytically validated assay Established management guidelines for prevention, diagnosis [^2^](#_ENREF_2) | confers cancer susceptibility [^3^](#_ENREF_3); also Category 2 with preclinical and emerging clinical data for DNA damaging agent therapy [^4^](#_ENREF_4)^,^ [^5^](#_ENREF_5) | member of research team to treating clinician and patient | Single child, no offspring.  Participant had responded to platinum-based therapy, but became resistant; commenced on Mitomycin C in third line. |
| Deleterious *ATM* germline mutation | | c.7271T>G (p.Val2424Gly) | Reported in HGMD as disease causing [^6^](#_ENREF_6)  Reported in KConfab database as pathogenic [^7^](#_ENREF_7) | confers cancer susceptibility [^8^](#_ENREF_8)^,^ [^9^](#_ENREF_9) | member of research team to treating clinician | Participant opted not to act. |
| Deleterious *BRCA2* germline mutation | | c.5722_5723delCT | Reported in the Breast cancer Information Core [BIC] database where it is listed as being clinically important [^1^](#_ENREF_1) Analytically validated assay Established management guidelines for prevention, diagnosis [^2^](#_ENREF_2) | confers cancer susceptibility [^3^](#_ENREF_3) | member of research team to treating clinician and patient | Not actioned, no further contact (participant died soon after written communication dispatched) |
| Category 2: Potential Clinical Significance | | | | | | |
| *BRAF (V600E)* mutation, *KRAS* wildtype, *APC* LOH | | *BRAF p.V600E* mutation, *KRAS* wildtype, *APC* LOH | Outlier according to mutational signature [^10^](#_ENREF_10)^,^ [^11^](#_ENREF_11) | Diagnostically relevant as clinical diagnosis was metastatic pancreatic cancer | member of research team to treating clinician | Further investigation to diagnose colorectal cancer rather than pancreatic |
| Mutational spectrum not consistent with neuroendocrine tumour | | Mutational Signature | Outlier according to mutational signature [^12^](#_ENREF_12) | Diagnostically relevant as clinical diagnosis was a pancreatic neuroendocrine tumour | member of research team to treating clinician | Diagnosis altered after further investigation and influenced treatment |
| Pancreatic mass in participant with metastatic melanoma | | *KRAS* G12D mutation,  *BRAF* wt | Outlier according to mutational signature [^10^](#_ENREF_10) | Diagnostically relevant, and required for clinical trial eligibility | member of research team to treating clinician | Diagnosed as pancreatic cancer based on genes mutated. |
| *BRCA2* somatic mutation | | c.5351dup  (p.Asn1784Lysfs*3) | Therapeutic opportunity  Evidence in scientific literature [^13^](#_ENREF_13) | Literature supporting potential activity of platinum agents in association with BRCA2 mutation[^13^](#_ENREF_13) | member of research team to treating clinician | Patient already responding to platinum-based therapy |
| Gemcitabine response in patient-derived xenograft | | NA | Clinically valid  Evidence in scientific literature [^14^](#_ENREF_14) | Pre-clinical data | member of research team to treating clinician | Patient opted to commence therapy based on finding |
| *KRAS* wild type | | NA | Therapeutic opportunity  Evidence in scientific literature [^15^](#_ENREF_15) | Clinical Trial Open | member of research team to treating clinician | disease free, no action to date |
| *KRAS* wild type | | NA | Therapeutic opportunity  Evidence in scientific literature [^15^](#_ENREF_15) | Clinical Trial Open | member of research team to treating clinician | lost to follow up |
| *KRAS* wild type | | NA | Therapeutic opportunity  Evidence in scientific literature [^15^](#_ENREF_15) | Clinical Trial Open | member of research team to treating clinician | eligible for clinical trial, recruited but withdrew |
| *KRAS* wild type | | NA | Therapeutic opportunity [^15^](#_ENREF_15)  Evidence in scientific literature | Clinical Trial Open | member of research team to treating clinician | eligible for clinical trial; not actioned to date (participant disease free) |
| *ERBB2 (HER2/neu)* Amplification | | NA | Therapeutic opportunity [^16^](#_ENREF_16)  Evidence in scientific literature [^17^](#_ENREF_17) | Potential for last line therapy choice | member of research team to treating clinician | Preference of clinician not to action findings |
| *ERBB2 (HER2/neu)* Amplification | | 10 or more HER2 gene copies per cell | Therapeutic opportunity [^16^](#_ENREF_16)  Evidence in scientific literature [^17^](#_ENREF_17) | Clinical Trial Open | member of research team to treating clinician | Preference of clinician not to action findings |
| *ERBB2 (HER2/neu)* Amplification | | 10 or more HER2 gene copies per cell | Therapeutic opportunity [^16^](#_ENREF_16)  Evidence in scientific literature [^17^](#_ENREF_17) | Clinical Trial Open | member of research team to treating clinician | not actioned - patient too fragile |
| *ERBB2 (HER2/neu)* Amplification | | 10 or more HER2 gene copies per cell | Therapeutic opportunity [^16^](#_ENREF_16)  Evidence in scientific literature [^17^](#_ENREF_17) | Clinical Trial Open | member of research team to treating clinician | eligible for clinical trial, not actioned to date (participant disease free) |
| Results returned on clinician request with no actionable phenotypes | | | | | | |
| Request results that may influence therapy | |  |  | Clinical Scenario | member of research team to treating clinician | Negative results returned for 8 participants |

**References**

**1.** Breast Cancer Information Core <http://research.nhgri.nih.gov/bic/>, 2010.

**2.** EviQ: Cancer Treatment Information Online https://<http://www.eviq.org.au/Home.aspx>.

**3.** Ford D, Easton DF, Stratton M, et al. Genetic heterogeneity and penetrance analysis of the BRCA1 and BRCA2 genes in breast cancer families. The Breast Cancer Linkage Consortium. *Am J Hum Genet.* Mar 1998;62(3):676-689.

**4.** Xia B, Dorsman JC, Ameziane N, et al. Fanconi anemia is associated with a defect in the BRCA2 partner PALB2. *Nature Genetics.* Feb 2007;39(2):159-161.

**5.** Villarroel MC, Rajeshkumar NV, Garrido-Laguna I, et al. Personalizing cancer treatment in the age of global genomic analyses: PALB2 gene mutations and the response to DNA damaging agents in pancreatic cancer. *Mol Cancer Ther.* Jan 2011;10(1):3-8.

**6.** Stenson PD, Mort M, Ball EV, Shaw K, Phillips AD, Cooper DN. The Human Gene Mutation Database: building a comprehensive mutation repository for clinical and molecular genetics, diagnostic testing and personalized genomic medicine. *Human genetics.* Sep 28 2013.

**7.** KConfab: Kathleen Cunnigham Consortium for Familial Breast Cancer. <http://www.kconfab.org/Progress/Mutations.aspx>, 2011.

**8.** Goldgar DE, Healey S, Dowty JG, et al. Rare variants in the ATM gene and risk of breast cancer. *Breast cancer research : BCR.* 2011;13(4):R73.

**9.** Bernstein JL, Teraoka S, Southey MC, et al. Population-based estimates of breast cancer risks associated with ATM gene variants c.7271T>G and c.1066-6T>G (IVS10-6T>G) from the Breast Cancer Family Registry. *Hum Mutat.* Nov 2006;27(11):1122-1128.

**10.** Biankin AV, Waddell N, Kassahn KS, et al. Pancreatic cancer genomes reveal aberrations in axon guidance pathway genes. *Nature.* Nov 15 2012;491(7424):399-405.

**11.** Network CGA. Comprehensive molecular characterization of human colon and rectal cancer. *Nature.* Jul 19 2012;487(7407):330-337.

**12.** Alexandrov LB, Nik-Zainal S, Wedge DC, et al. Signatures of mutational processes in human cancer. *Nature.* Aug 22 2013;500(7463):415-421.

**13.** Showalter SL, Charles S, Belin J, et al. Identifying pancreatic cancer patients for targeted treatment: the challenges and limitations of the current selection process and vision for the future. *Expert Opin Drug Deliv.* Mar 2010;7(3):273-284.

**14.** Nakano Y, Tanno S, Koizumi K, et al. Gemcitabine chemoresistance and molecular markers associated with gemcitabine transport and metabolism in human pancreatic cancer cells. *British journal of cancer.* Feb 12 2007;96(3):457-463.

**15.** Luo G, Long J, Qiu L, Liu C, Xu J, Yu X. Role of epidermal growth factor receptor expression on patient survival in pancreatic cancer: a meta-analysis. *Pancreatology.* 2011;11(6):595-600.

**16.** Chou A, Waddell N, Cowley MJ, et al. Clinical and molecular characterization of HER2 amplified-pancreatic cancer. *Genome medicine.* Aug 31 2013;5(8):78.

**17.** Harder J, Ihorst G, Heinemann V, et al. Multicentre phase II trial of trastuzumab and capecitabine in patients with HER2 overexpressing metastatic pancreatic cancer. *British journal of cancer.* Mar 13 2012;106(6):1033-1038.
