# Supplementary material for: Returning individual research results for genome sequences of pancreatic cancer
Source: Genome Med. 2014 May 29;6(5):42. doi: 10.1186/gm558 (PMC4067993; doi:10.1186/gm558)
Supplement: Additional file 2 — All associated approving human research ethics committees. [file gm558-S2.docx]

**Supplementary File 2: Details of Approving Human Research Ethics Committees**

- Sydney South West Area Health Service Human Research Ethics Committee, Western Zone, protocol number 2006/54
- Sydney Local Health District Human Research Ethics Committee, protocol number X11-0220
- Northern Sydney Central Coast Health Human Research Ethics Committee, protocol number 0612-251M
- Sydney West Area Health Service Human Research Ethics Committee (Westmead Campus), protocol number HREC2002/3/4.19
- South East Sydney Illawarra Area Health, Northern Hospital Network HREC- protocol number 05/321
- South East Sydney Illawarra Area Health HREC- Southern Section, protocol number 05/54
- St John of God Hospitals Subiaco & Murdoch:  385
- Fremantle Hospital:  09/324
- Royal Adelaide Hospital:  091107a
- Flinders Private Hospital:  167/10
- Austin Hospital:  H2011/04083
- Princess Alexandra Hospital:  09/QPAH/220
- Greenslopes Private Hospital:  09/34
