# Supplementary material for: Returning individual research results for genome sequences of pancreatic cancer
Source: Genome Med. 2014 May 29;6(5):42. doi: 10.1186/gm558 (PMC4067993; doi:10.1186/gm558)
Supplement: Additional file 3 — APGI Return of Results Guidelines, which outline standard procedures instituted for the return of results process. [file gm558-S3.pdf]

Standard Operating procedure: Guidelines on Returning Individual Research Results (RoR) to Individuals

1. Background

Over the last decade, next-generation sequencing technology (NGS) has developed to the point that rapid and relatively affordable sequencing of individual genomes is a reality. NGS offers the promise of tremendous public benefit as it underpins both improvements in our understanding of disease as well as substantially changing our practical ability to translate this knowledge through improved diagnostics and therapeutics. The advent of NGS has also seen a parallel increase in the debate around whether and how to disclose individual results of genetic sequencing to research participants. Whether researchers have a moral obligation to provide findings of this nature back to research participants is a vexed issue with no international consensus. The matter is complicated by the fact that unlike clinically validated genetic tests, WGS and other high throughput research techniques are research tools – they are not designed for clinical diagnosis and they may produce results that are of questionable clinical utility. Results from these analyses may identify genetic variants that are related to increases in disease risk, but the increased risk may be exceptionally small. Nevertheless, findings that are currently uncertain may, in future, become clinically relevant, and on this basis that the APGI has considered this issue at length, and conlcuded that within the context of a robust framwework and close consulation with clincial teams, returning results to participants is feasible and should be conducted whenever indicated.

2. Key RoR Principles

- Important research results are often referred to under various designations, such as incidental or secondary findings, off target results etc, and are used interchangebly throughout literature and policy.
- These guidelines and all policies and procedures related to RoR are approved and goverend by the APGI leadership team (http://www.pancreaticcancer.net.au/apgi/leadership-teams).
- The point of policy reference for these guideliens is NHMRC National Statement on Ethical Conduct in Human Resaerch 2007.
- It is crucial that each result is assessed on a case by case basis, carefully considering context along with the significance of the result.
- Managing RoR is a shared responsibility between researchers and clinical teams, and at all times where appropriate researchers should work with clincial teams to ensure information is handled appropriately and in-line with the ethically defensible plan described herein.
- RoR is an opt-in situation for the APGI participants, which is addressed thorugh the consent process and recorded thorugh CanSto_Pancreas.

- These guidelines are not all-encompassing, as every situation that arises will be varied. Procedures presented herein are designed to be iterative and updated every 6 months (or at every 2nd ICGC data release).

3. Ethically Defensible Plan

The Australian National Statement on ethical conduct in Human Research (2007) is designed to clarify repsonsibilityes of insitutions and resaerchers for the ethical design, conduct and dissemination of results of human research. Return of results is addressed in section 3.5.1, where it states: “Where research may discover or generate information of potential importance to the future health of participants, or their blood relatives, researchers must prepare and follow an ethically defensible plan to disclose or withhold that information”. The APGI has developed an Ethically Defensible Plan (EDP), which has been approved by all HREC’s at active APGI sites. The EDP framework set out by the APGI for returning results employs a context-dependant paradigm, and enacts a broad category-based system for the characterisation of research findings. The framework was developed as an iterative, evidence-based and consensus driven process, with engagement of key stakeholders. As the primary resaearch indication is the interrogation of cancer genomes for the discovery and analysis of driver mutations, the return of results is lmited in most cases to findings related to cancer.

The key components of the plan are:

1. Consent

Patients would have the option of being notified of a clinically relevant research finding, which would be conveyed through the informed consent process under the section "What if Something is Found". Due to the aggressive nature and poor survival of pancreatic cancer, we may need to include nominated family members of participants in this process. This is also outlined in the Information Statement and Consent Form in the section “What if Something is Found”. These preferences are tracked and logged through the CanSto_Pancreas Database.

2. Significance of Results

A broad based categorical system is enacted, based on the below:

a. ***Medically Actionable Findings***: where findings represent directly utility, in terms of disease prevention, or where a medical benefit exists of a prognotic, diganostic or therpautic nature. These types of findings often have estbalished treatment guidelines and confer a high risk of a preventbale disease. These are often referred to as *analytically valid* findings. The majortity of results faling into this category are germline suspectibility mutations, usch as mutation in the *BRCA1/2* or *ATM* genes. This category also exemplifies somatic mutation inofmration that may indicate a discordant pathology

diagnosis. These findings are always confirmed in an indpeandant diagsnotic grade assay.

b. ***Findings of poteintal clinical utility***: these findings have demostrated clinical validity, but lack demostrated clinical utility in a current reatment setting. They also may be members of cancer pathways or fnctional groups that are targets for approved or investigational therpeautic agents or biomarkers.

c. Findings of undetermined significance: these variants are not reoutinely returned and form the focus of on-going research.

3. Communicability & delivery of results

Communicability considers the practicality of communicating results, the circumstances of the patient and treating clinician, while delivery of results considers how best the results could be communicated. This is an important pint to consider in sensitive situations such as particpants being deceased.

This ethically defensible plan is based on beneficence, reciprocity and respect for participants, which are all fundamental ethical principles in research. On contact by the treatment team, participants have the option not to pursue further information, which values the autonomy and voluntary nature of research participation.

3. Procedure

The below flow chart represents the overall process that is to be generally followed.

Result uncovered warranting return where patient has indicated preference to receive results

Does the result represents clinically useful or medically actionable information as deemed by the guidelines set out in the EDP?

YES

Follow Confirmatory Testing SOP Guidelines to establish analytical validity via diagnostic grade assay

✓ Result validates

Does not validate ✗

Table discrepancy at Molecular MDT

Proceed to return result

ACTIONABLE PHENOTYPE

Result is of diagnostic importance or has therapeutic potential

CONTACT ESTABLISHED WITH TREATMENT TEAMS

Medical oncologist and/or Surgeon

NOTIFICATION LETTER COMPLETED

by the Project Manager and signed by the PI

- ☑ Document copied to other relevant medical teams
- ☑ Document uploaded into database

LETTER ISSUED TO PRIMARY CLINICIAN

- ☑ Relevant confirmatory testing report attached
- ☑ Any clinical trial details explained
- ☑ Event recorded in database

CLINICIAN DISCUSSES RESULT WITH PATIENT

if the clinician deems the result to be relevant

FOLLOW UP OF PATIENT

resumes as per APGI standard protocol

GERMLINE SUSCEPTIBILITY MUTATION

Result has significance for family members

STATUS OF PATIENT CONFIRMED

Alive

NOTIFICATION LETTER COMPLETED

by the Project Manager and signed by the PI

- ☑ Document copied to other relevant medical teams
- ☑ Document uploaded into database

LETTER ISSUED TO PRIMARY CLINICIAN

- ☑ Relevant confirmatory testing report attached
- ☑ Any clinical trial details explained
- ☑ Event recorded in database

CLINICIAN DISCUSSES RESULT WITH PATIENT

RESEARCH TEAM COMPLETES REFERRAL TO FAMILIAL CANCER CLINIC (if required)

Referral to Prince of Wales FCC if patient lives locally or contact made with relevant clinic in nearest hospital

- ☑ Relevant documents attached (consent form, pathology report, treatment summary etc.)
- ☑ Document uploaded into database

Deceased

NOTIFICATION LETTER ISSUED TO DOCUMENTED NOK OR SIGNIFICANT OTHER

NOK and partners are encouraged to contact research team for a referral to a Familial Cancer Clinic

If research team is contacted

REFERRAL TO FAMILIAL CANCER CLINIC DRAFTED

- ☑ Relevant documents attached (consent form, pathology report, treatment summary etc.)
- ☑ Document uploaded into database

As this represents a sensitive situation, the research team should make verbal contact with the FCC prior to sending referral

REFERRAL SENT TO FAMILIAL CANCER CLINIC

- ☑ Event recorded in database

CLINIC MAKES CONTACT WITH PATIENT VIA STANDARD INTAKE PROCEDURES

Clinic is encouraged to copy Project Manager into any forthcoming correspondence
