# Supplementary material for: Returning individual research results for genome sequences of pancreatic cancer
Source: Genome Med. 2014 May 29;6(5):42. doi: 10.1186/gm558 (PMC4067993; doi:10.1186/gm558)
Supplement: Additional file 4 — Standard language used in the Participant Information Statement. [file gm558-S4.docx]

Supplementary File 4: Example Wording of Participant Information Statement and Consent Form

1. What you should know about this research study:

You are invited to take part in this research project. The research project is aiming to learn about the reasons people develop tumours and cancers of the pancreas and how they may best be treated. People who have a pancreatic tumour or cancer now, or those believed to have a tumour may join this research project.

This Participant Information and Consent Form tells you about the research project. It explains the procedures involved. Knowing what is involved will help you decide if you want to take part in the research.

Please read this information carefully. Ask questions about anything that you don’t understand or want to know more about. Before deciding whether or not to take part, you might talk about it with a relative, friend or healthcare worker. We recommend you discuss your potential involvement in this study with your family members.

Participation in this research is voluntary. If you don’t wish to take part, you don’t have to. You will receive the best possible care whether you take part or not.

If you decide you want to take part in the research project, you will be asked to sign the consent section. By signing it you are telling us that you:

- Understand what you have read
- Consent to take part in the research project
- Consent to participate in the processes that are described
- Consent to the use of your personal and health information as described

You will be given a copy of this Participant Information and Consent Form.

2. Why is this research being done?

This research is being done to learn about the reasons people develop tumours and cancers of the pancreas and how they may best be treated. We are also hoping to learn how these tumours develop, and how tumours of the pancreas differ from normal cells. We will do this by studying the genes (DNA, RNA) and proteins in pancreatic cancers and normal cells. Our goal is to identify which people will develop tumours and cancers of the pancreas in the future, to develop better ways to screen for early tumours, and to develop future treatments for pancreatic cancer.

We hope to study about 100 participants each year (for a total of 500)*.* Patients from all over Australia will be invited to participate in the study. It is part of a larger research study known as the International Cancer Genome Consortium (ICGC). The ICGC is an international effort to coordinate a large number of research projects aimed at establishing a comprehensive catalogue of the genetic changes that are present in many types of cancer. It is hoped that both new detection strategies as well as new treatments may be developed from these discoveries.

3. What does participation in this research project involve?

If you agree to be in this study, we will ask you to do the following things:

1. We will ask you to tell us about your own and family’s health background. To do this we may ask you to participate in a short questionnaire, and allow us to obtain your relevant medical records from other doctors or hospitals. We would also like to access records held about you at the New South Wales Cancer Registry.
2. Give us a blood sample. In most cases this will be done as part of your routine testing. We will collect 3 vials of blood, totalling 20mL.
3. Allow us to collect normal and abnormal tissue samples taken during the diagnosis and treatment of your condition. This may include Electronic Retrograde Cholangio-Pancreatography (ERCP), Endoscopic Ultrasound (EUS), biopsy or surgery. This does not require the removal of extra tissue. It will not involve any extra risk or discomfort to you; it means that you give us permission to take some of the sample already taken for medical reasons, for our research.
4. Allow us to collect tissue samples that have been previously collected from you. We want to study any extra tumour, cancer, normal tissues, secretions or other samples collected as part of your regular treatment. We may need to be able to grow and treat your tumour cells under special laboratory conditions as a necessary part of this study.
5. Let us store your samples for future research studies related to our findings in this study.
6. Allow Garvan Institute research staff to contact you periodically to see how you are doing, so that we can look at how the genes, proteins or factors in your tumour relate to survival. Not everyone who gives permission will be contacted and you can decline to be re-contacted if you wish. You can still participate in this study if you declined to be re-contacted.
7. Allow us to share your coded data, including coded biological samples with investigators at other institutions conducting studies with our research team, aimed to allow us to better understand the causes of pancreatic cancer. In particular we will share samples and information with investigators in the International Cancer Genome Consortium (ICGC). The samples will not contain any personal information. We have rules in place to protect your privacy. These are outlined later in this form.

**4. What will happen to my samples?**

Your tissue and other samples, the data derived from any analyses of those samples and your personal information found in your health records will be coded to protect your confidentiality. Your samples will be sent to the Garvan Institute of Medical Research in Sydney, NSW where staff will remove personal identifiers, such as your name and address, and replace them with a unique code. This unique code will enable us to link the information from different datasets, for example, your medical records, to your samples. Researchers will only receive coded information and will not have access to your identity. This system will enable us to keep your identity confidential. Specifically, we will keep your personal details separate from your coded data through computers dedicated to this project and use stringent security measures to prevent unauthorized use, including: strict access controls, computer security and data encryption techniques, confidentiality agreements and staff training.

Your tissue and blood samples will be used for this research, and for other extra tests related to our findings in this study. Your samples may also be shared for approved studies between other investigators conducting studies with our research team in Australia, or among ICGC members. The Garvan Institute will be the custodian of your samples, and they will be stored here until they are used entirely by this project.

We will be able to re-link your personal details with your coded data, but this will only be done in order to make sure the database records are correct or in case you want to withdraw from the study.

Your samples and information will not be released for other uses without your prior consent, unless required by law.

**5. What if something is found?**

As part of this research study, your samples may be used to look for genes, proteins or factors which put people at risk for tumours or cancers, not limited to pancreatic cancer. Generally, we will not tell you the results of studies done on your samples. However, if we learn something in the future that may have significance to you or your family in regards to cancer you can have the choice to be notified of this information. In such instances we would first communicate the information to your treating doctor.

In case you are not available we may need to contact your nominated family member or designee provided on page 6.

**6. How long will my tissue and information be in this study?**

Your tissue and information will be in this study for an indefinite period of time.

7. What are the possible benefits?

There may be no direct benefit to you from being in this study. Knowledge from this study may help treatment of people with pancreatic cancer and other tumours in the future.

8. What are the possible risks?

Taking blood may cause some discomfort, bleeding or bruising when the needle enters the body, these can be easily treated. There is a very small risk of infection.

You may get tired or bored when we are asking you questions or you are completing questionnaires. You do not have to answer any questions you do not want to answer.

There may be additional risks that the researchers do not expect or do not know about. Tell a member of the research team immediately about any new or unusual symptoms that you get.

9. What if new information arises during this research project?

During the research project, new information about the risks and benefits of the project may become known to the researchers. If this occurs, you will be told about this new information and your doctors will discuss whether this new information affects you.

10. Are there alternatives to participation?

You do not have to participate in this study. If you do not participate, the care at your treating hospital will not be affected.

11. Do I have to take part in this research project?

Participation in any research project is voluntary. If you do not wish to take part you don’t have to. If you decide to take part and later change your mind, you are free to withdraw at any stage.

Your decision whether to take part or not to take part, or to take part and then withdraw, will not affect your routine treatment at your treating hospital.

- What will happen to information about me?

Any information obtained in connection with this research project that can identify you will remain confidential and will only be used for the purpose of this research project. It will only be disclosed with your permission, except as required by law.

Information about you may be obtained from your health records held at this, and other, health services for the purposes of this research.

We plan to discuss results of this research at meetings of scientists and doctors, and publish the results in medical journals. However in any publication or discussion, we will write the results so that you cannot be identified.

- How can I access my information?

In accordance with relevant Australian and/or New South Wales privacy and other relevant laws, you have the right to access the information collected and stored by the researchers about you. You also have the right to request that any information, with which you disagree, be corrected. Please contact the Principal Investigator listed in this document if you would like to access your information.
